# Esophageal squamous cell carcinoma with low mitochondrial copy number has mesenchymal and stem-like characteristics, and contributes to poor prognosis

**DOI:** 10.1371/journal.pone.0193159

**Published:** 2018-02-15

**Authors:** Yasunori Masuike, Koji Tanaka, Tomoki Makino, Makoto Yamasaki, Yasuhiro Miyazaki, Tsuyoshi Takahashi, Yukinori Kurokawa, Kiyokazu Nakajima, Masaki Mori, Yuichiro Doki

**Affiliations:** Department of Gastroenterological Surgery, Graduate School of Medicine, Osaka University, Osaka, Japan; Baylor College of Medicine, UNITED STATES

## Abstract

Alterations in mitochondrial DNA (mtDNA) copy numbers in various human cancers have been studied, but any such changes in esophageal squamous cell carcinoma (ESCC) are not established. In the present study, we investigated the correlation of mtDNA copy number with clinicopathologic features, prognosis, and malignant potential of ESCC. MtDNA copy numbers of resected specimens from 80 patients treated with radical esophagectomy were measured by quantitative real-time PCR analyses. Human ESCC cells, TE8 and TE11, were cultured, and depletion of mtDNA content was induced by knockdown of mitochondrial transcription factor A expression or treatment with ethidium bromide. The mRNA and protein expression, proliferation, invasion, and cell cycle were investigated. The results showed that the mtDNA copy number of cancerous portions was 56.0 (37.4–234.5) percent that of non-cancerous parts and significantly lower (p<0.01). Low mtDNA copy number in resected cancerous tissues was significantly correlated with pathological depth of tumor invasion (p = 0.045) and pathological stage (p = 0.025). Patients with lower mtDNA copy number had significantly poorer 5-year overall survival compared to patients with higher levels (p<0.01). The mtDNA-depleted TE8 and TE11 cells had morphological changes and proliferated more slowly than control cells under normoxia but proliferated at almost the same rate under hypoxic conditions. In mtDNA-depleted cells, E-cadherin mRNA expression was decreased, and N-cadherin, vimentin, zeb-1, and cd44 mRNA expression was increased. Immunoblotting and flow cytometry analysis also showed downregulated E-cadherin and upregulated N-cadherin and CD44 protein in mtDNA-depleted cells. Moreover, mtDNA-depleted cells had enhanced invasion, migration, and sphere formation abilities, and the cell cycle arrest at G0/G1 phase was induced in these cells. These results suggested that mtDNA-depleted ESCC cells had mesenchymal characteristics, cancer stemness, and tolerance to hypoxia, which played important role in cancer progression. In conclusion, a low copy number of mtDNA is associated with tumor progression in ESCC.

## Introduction

Esophageal cancer is the eighth most common cancer worldwide, with an estimated 450,000 new cases annually, and the sixth most common cause of death from cancer, with an estimated 400,000 deaths each year. The incidence rate is highest in Eastern Asia, where the dominant histological subtype is squamous cell carcinoma [1–4]. The combination therapies of preoperative chemotherapy with or without radiotherapy followed by surgery have been developed and widely implemented as effective treatments for advanced esophageal squamous cell carcinoma (ESCC) [5–8]. However, especially in more advanced cases, the survival outcome is poor [9, 10]. To improve the prognosis of ESCC, new therapeutic targets are required.

Mitochondria are eukaryotic intracellular organelles that produce the majority of cellular ATP through the process of oxidative phosphorylation, and also play an important role in reactive oxygen species production and integrating apoptosis pathways [11–13]. They also contain their own DNA (mtDNA), which consists of a circular double-stranded structure with 16,569 base pair and encodes 13 polypeptides that are essential for the assembly of respiratory enzyme complexes [12, 14–16]. Each human cell contains several hundreds to thousands of mitochondria [14, 17, 18]. Alterations in mtDNA copy numbers in various human cancers have been studied in the past few decades [19–25], but the mtDNA copy number and its significance in ESCC remain unclear. The aim of this study was to clarify the correlation of mtDNA copy number with clinicopathologic features, prognosis, and cell characteristics of ESCC.

## Materials and methods

### Clinical samples

Formalin-fixed, paraffin-embedded samples were collected from 80 patients with ESCC who had undergone surgical resection without neoadjuvant therapy at Osaka University Hospital (Osaka, Japan) between April 2002 and July 2014. Tumor stage was classified according to the 8^th^ edition of the American Joint Committee on Cancer and The Union for International Cancer Control (AJCC/UICC) staging system [26]. Using laser microdissection by LMD7000 (Leica Microsystems, Wetzlar, Germany), 80 cancerous ESCC nests were subjected to DNA extraction. From 20 patients, non-cancerous samples were also obtained and subjected to DNA extraction. All patients provided written informed consent regarding the use of the resected specimens, and this study was approved by the ethics committee of Osaka University, Graduate School of Medicine (Permit number #15401).

### Cell culture and mtDNA depletion

Human ESCC cells, TE8 (RBRC-RCB2098) and TE11 (RBRC-2100), were purchased from the RIKEN BioResource Center (Tsukuba, Ibaraki, Japan). The cells were cultured in RPMI-1640 medium supplemented with 10% fetal bovine serum (Thermo Fisher Scientific, Waltham, MA, USA), penicillin (100 IU/ml), and streptomycin (100 μg/ml) at 37°C in a humidified incubator with 5% CO_2_. Depletion of mtDNA content was induced by knockdown of mitochondrial transcription factor A (TFAM) expression or treatment with ethidium bromide (EtBr, 100 ng/ml) for 4 passages [27–30]. Because TFAM is critical for mtDNA packaging and maintenance [31], silencing this factor reduces mtDNA content. A short hairpin RNA designed by Sigma-Aldrich (St. Louis, MO, USA), MISSION TRC-Hs1.0, was applied for knockdown of TFAM expression. The target sequences against TFAM gene were 5’-CGTCGCACAATAAAGAACAA-3’ (TRCN0000016094, defined as ‘tfam-sh1’) and 5’-GCAGATTTAAAGAACAGCTAA-3’ (TRCN0000016097, defined as ‘tfam-sh2’). For comparison, a non-target sequence of 5’-GGCGCGATAGCGCTAATAATTT-3’ (SHC016, Sigma-Aldrich, defined as ‘control-sh’) was used as the control. The cells treated by EtBr were defined as ‘EtBr’. The cells with reduced mtDNA copy number were cultured in the medium with 1 mM sodium pyruvate and 50 μg/ml uridine [27, 29]. The morphology of the cells was examined by optical microscopy (BZ-X710, KEYENCE, OSAKA, Japan).

### Measurement of mtDNA copy number and mRNA expression levels

Genomic DNA from cancerous and non-cancerous tissues was extracted using the GeneRead DNA FFPE Kit (180134, Qiagen) according to the manufacturer’s instruction. The mtDNA copy number was measured by quantitative real-time PCR using specific primers for mtDNA-coded *Cytochrome Oxidase I* (Forward;5’-TGATCTGCTGCAGTGCTCTGA-3’, Reverse; 5’-TCAGGCCACCTACGGTGAA-3’) and nuclear DNA–coded *Cytochrome Oxidase IV* (Forward;5’-GAAAGTGTTGTGAAGAGCGAAGAC-3’, Reverse;5’-GTGGTCACGCCGATCCAT-3’) genes after adjusting with the mtDNA copy number of the TE11 cell as 1.00 [27, 29]. The mRNA expression levels of mtDNA-depleted cells were measured by quantitative RT-PCR and normalized to the expression of *β-actin* and compared with control-sh cells or parental cells as 1.00.

### Lactate assay

A total of 2.0×10^5^ cells per well were seeded in 6-well plates. Extracellular L(+)-lactate in culture medium was detected using a lactate assay kit (MAK064, Sigma-Aldrich) after incubation for 48 h, following the manufacturer’s instructions.

### Transmission electron microscopy

A total of 1.0×10^5^ cells per well were seeded in 6-well plates and cultured at 37°C for 48 h, followed by fixation in 2.5% glutaraldehyde. The fixed cells were embedded in resin with Quetol-812 (Nissin EM, Tokyo, Japan) and cut into ultrathin (80 nm) sections using the Reichert-Jung Ultracut E (Reichert, Vienna, Austria). Finally, the stained ultramicrotomies were observed and imaged with a transmission electron microscope (H-7650, Hitachi, Tokyo, Japan).

### Cell proliferation and viability assay

A total of 4.0×10^3^ cells per well were seeded in 96-well plates, and cell viability was assessed using the Cell Counting kit-F (CK06, Dojindo, Japan) at 24 h, 48 h, and 72 h after incubation under normoxia (20% O_2_) or hypoxia (1% O_2_). The hypoxia-resistant rate was assessed by the ratio of the proliferation rate under hypoxia to that under normoxia at each time point. The fluorescence intensity was measured by SH-9000lab (Corona Electric, Ibaraki, Japan) on a plate reader at an excitation wavelength of 490 nm and emission wavelength of 515 nm.

### Immunoblotting analysis

The relative protein expression levels were investigated by immunoblotting. TFAM, E-cadherin and N-cadherin expression levels were monitored using a commercially available anti-TFAM rabbit polyclonal antibody (1:1000 dilution, ab47517, Abcam, Cambridge, UK), anti-E-cadherin mouse monoclonal antibody (1:500 dilution, 610181, BD Biosciences), anti-N-cadherin mouse monoclonal antibody (1:500 dilution, 610920, BD Biosciences) and anti-actin rabbit polyclonal antibody (1:1000 dilution, A2066, Sigma-Aldrich).

### Flow cytometry analysis

The relative protein expression levels of CD44 were investigated by flow cytometry. Harvested cells were stained with an APC conjugated anti-CD44 antibody (4103011, BioLegend, San Diego, CA, USA). Flow cytometry was carried out using FACSCantoⅡ (BD Biosciences) and analyzed by FlowJo ver 10.3 (TOMY DIGITAL BIOLOGY, Tokyo, Japan), and mean fluorescence intensities (MFI) were measured.

### Invasion assay

To measure the invasive conditions of cells, we used 24-well inserted plates with 8 μm membrane pores and Matrigel coating (#354480, Corning, NY, USA). A total of 1.0×10^5^ (TE8) or 5.0×10^4^ (TE11) cells were added to the upper chamber, and 0.75 ml medium was added in the lower chamber. After cells were incubated at 37°C for 48 (TE8) or 24 h (TE11), non-invasive cells in the top chamber were removed by cotton swabs. Invasive cells at the bottom of the membrane were fixed and stained with the Diff-Quick stain kit (16920, Sysmex, Hyougo, Japan). Finally, the number of invasive cells was counted.

### Migration (scratch-wound healing) assay

Confluent monolayer of cells in 6-well plates were scratched using a pipette tip, and cells migrating into this area were observed under a microscope. Images were extracted at 0, 6, and 12 h after the scratch. For quantitative analysis, the wounded area was measured using ImageJ software [32, 33]. The wound-healing rate was assessed at 6 h and 12 h.

### Cell cycle assay

For cell cycle analysis, harvested cells were washed with PBS and fixed with ice-cold 70% ethanol and then treated with RNase and stained with propidium iodide (PI). After staining, flow cytometry was carried out using FACSCantoⅡ (BD Biosciences) and analyzed by FlowJo ver 10.3 (TOMY DIGITAL BIOLOGY, Tokyo, Japan). The duration of each cycle was assessed based on the results of the cell proliferation assay.

### Sphere formation assay

Cells were resuspended in serum-free MEGM BulletKit medium (CC-3150, Lonza, Allendale, NJ, USA) and plated in ultra–low-attachment 96-well plates (#3474, Corning, NY, USA) at 200 cells per well. The BulletKit medium included bovine pituitary extract, epidermal growth factor, insulin, hydrocortisone, and antibiotics. The number of spheres in each well was counted at day 7.

### Statistical analysis

All analyses and experiments were repeated at least three times. Statistical analyses were performed with JMP^Ⓡ^ Pro 12 (SAS Institute Inc., Cary, NC, USA). Fisher’s exact test or a χ^2^ test was applied to evaluate the association between mtDNA copy number and clinicopathological factors. The recurrence-free survival (RFS) and overall survival (OS) rates of patients were estimated by Kaplan–Meier survival analysis and compared using the log-rank test. RFS was defined as the time from surgical resection to recurrence of the cancer or death from any cause. OS was defined as the time from surgical resection to death from any cause. The Cox’s proportional hazards regression model was performed to identify factors influencing the RFS of ESCC. Other statistical analyses were performed using Student’s t-tests. The difference was considered significant when a p value was less than 0.05. In the cell culture assay, ‘*’ and ‘**’ indicates a significant difference between tfam-sh1 and control-sh cells (*p value <0.05, **p value <0.01). ‘^†^’ and ‘^††^’ indicated significant difference between tfam-sh2 and control-sh cells (^†^p value <0.05, ^††^p value <0.01).

## Results

### The mtDNA copy number in ESCC cells is correlated with tumor progression and prognosis

**Fig 1:**
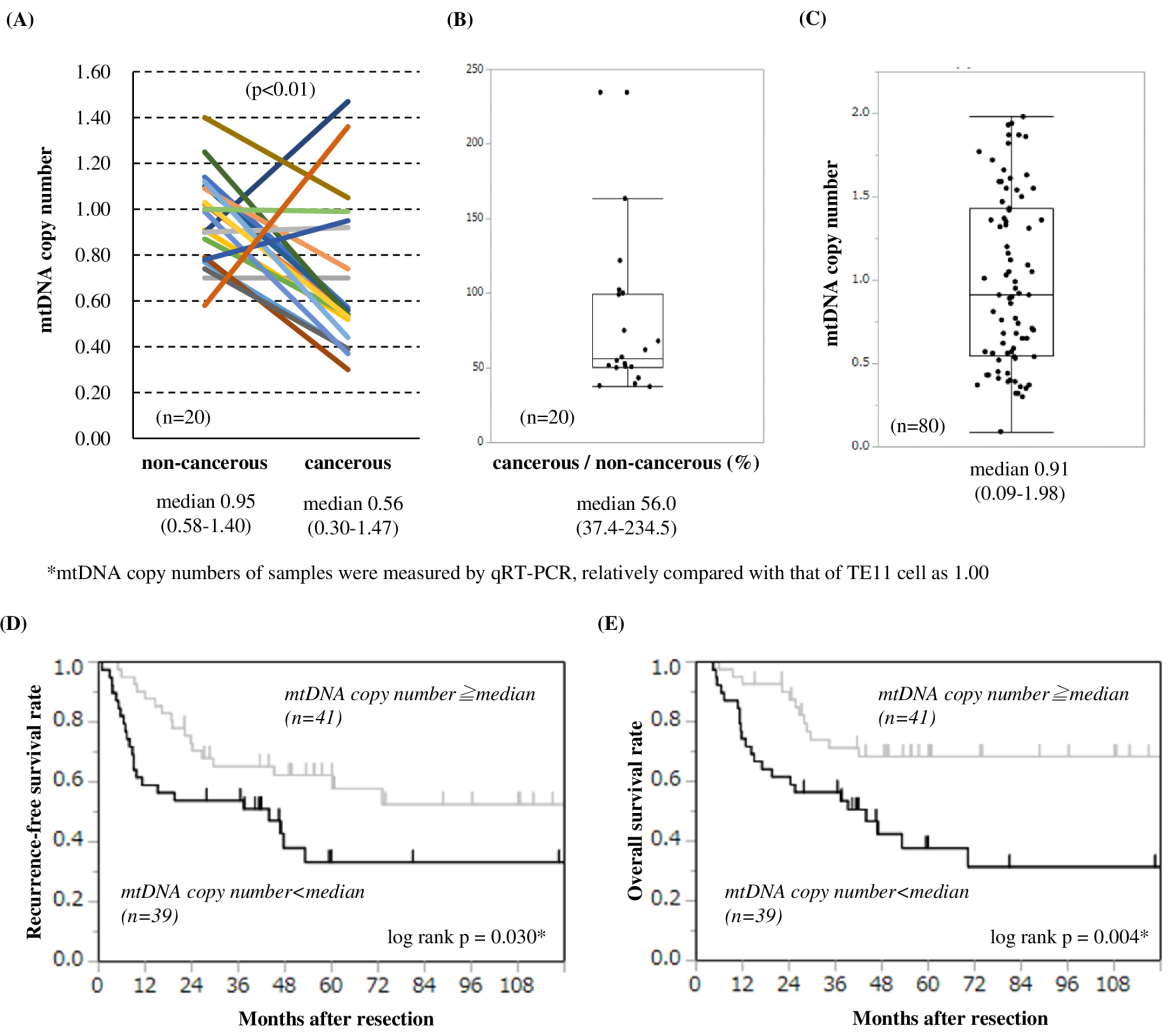
MtDNA copy number of the non-cancerous and cancerous samples and patient survival. **(A)** The mtDNA copy numbers of the cancerous samples from 20 patients were significantly lower than that of paired non-cancerous samples (median 0.56 (0.30–1.47) vs 0.95 (0.58–1.40), p<0.01). **(B)** The mtDNA copy number of the cancerous samples was 56.0 (37.4–234.5) percent of the non-cancerous parts. **(C)** The median mtDNA copy numbers of the ESCC cancerous samples from 80 patients was 0.91 (0.09–1.98), adjusting with the mtDNA copy number of the TE11 cell as 1.00. The Kaplan–Meier survival curve and the log-rank test were used to analyze the RFS **(D)** and OS **(E)** of patients according to mtDNA copy number levels. Patients with lower mtDNA copy number showed significantly lower rates of RFS and OS than the higher group (*p<0.05).

The median patient age was 71.5 years (range, 42–85 years). A total of 19 patients had clinical Stage I, 44 had clinical Stage II, 14 had clinical Stage III, and 3 had clinical Stage IV disease (Table 1). Compared to paired non-cancerous samples among 20 patients, the mtDNA copy numbers of the cancerous samples were significantly decreased (median 0.56 (0.30–1.47) vs 0.95 (0.58–1.40), p<0.01; Fig 1A). The mtDNA copy number of the cancerous samples was 56.0 (37.4–234.5) percent of the non-cancerous portion (Fig 1B). The median mtDNA copy number of ESCC samples from 80 patients was 0.91 (0.09–1.98) (Fig 1C).

**Table 1:** Patient characteristics.

Characteristic		Number of patients
Age	Median	71.5
	(range)	(42–85)
Sex	Male/Female	65 / 15
Location	Ut / Mt / Lt	13 / 32 / 35
Histology	wel / mod / por	19 / 32 / 9
cT	1 / 2 / 3 / 4	21 / 30 / 22 / 7
cN	0 / 1 / 2 / 3	62 / 17 / 1 / 0
cM	0 / 1	77 / 3
cStage	I / II / III / IV	19 / 44 / 14 / 3
pT	1 / 2 / 3 / 4	32 / 14 / 30 / 4
pN	0 / 1 / 2 / 3	36 / 25 / 12 / 7
pM	0 / 1	77 / 3
pStage	I / II / III / IV	28 / 21 / 28 / 3

wel, well-differentiated squamous cell carcinoma; mod, moderately differentiated squamous cell carcinoma; por, poorly differentiated squamous cell carcinoma; cT/cN/cM/cStage, clinical classification according to the 8^th^ AJCC/UICC TNM (tumor, node, metastases) classification; pT/pN/pM/pStage, pathological classification according to the 8^th^ AJCC/UICC TNM classification

Patients also were categorized into higher and lower groups based on the median mtDNA copy number of 0.91. Lower copy number was significantly correlated with high pathological tumor invasion and stage (pT, p = 0.045; pStage p = 0.025; Table 2). Moreover, the lower mtDNA copy number group was more likely to have venous invasion, but there was no significant correlation (p = 0.076).

**Table 2:** Relationship between mtDNA copy number and clinicopathological characteristics.

Characteristic	mtDNA copy number		p value
	Higher group	Lower group	
	n (%)	n (%)	
Age, years			0.476
<70	19 (46.3)	15 (38.5)	
≥70	22 (53.7)	24 (61.5)	
Sex			0.694
Male	34 (82.9)	31 (79.5)	
Female	7 (17.1)	8 (20.5)	
Histology			0.679
wel, mod	36 (87.8)	33 (84.6)	
por	5 (12.2)	6 (15.4)	
Lymphatic invasion			0.529
negative	12 (29.3)	9 (23.1)	
positive	29 (70.7)	30 (76.9)	
Venous invasion			0.076
negative	27 (65.9)	18 (46.2)	
positive	14 (34.1)	21 (53.8)	
pT			0.045*
0–2	28 (68.3)	18 (46.2)	
3–4	13 (31.7)	21 (53.8)	
pN			0.252
0	21 (51.2)	15 (38.5)	
1–3	20 (48.8)	24 (61.5)	
pM			0.586
0	39 (95.1)	38 (97.4)	
1	2 (4.9)	1 (2.6)	
pStage			0.025*
I-II	30 (73.2)	19 (48.7)	
III-IV	11 (26.8)	20 (51.3)	

pT/pN/pM/pStage, pathological classification according to the 8^th^ AJCC/UICC TNM (tumor, node, metastases) classification

*p<0.05

Survival analyses showed that ESCC patients with lower mtDNA copy number had significantly poorer 5-year RFS and OS (RFS: 33.3% vs 62.3%, p = 0.030; OS: 31.5% vs 68.4%, p<0.01) (Fig 1D and 1E). On univariate analysis of OS, pathological AJCC/UICC T (tumor size) and N (node involvement) factors and mtDNA copy number were significant predictors of survival (S1 Table). On multivariate analyses, independent predictive factors associated with decreased OS rate were pathological T stage (hazard ratio (HR) 2.301, 95% confidence interval (CI) 1.116–4.891, p = 0.024) and mtDNA copy number (HR 2.281, 95%CI 1.144–4.781, p = 0.019).

### Establishment of cells with decreased mtDNA copy number

**Fig 2:**
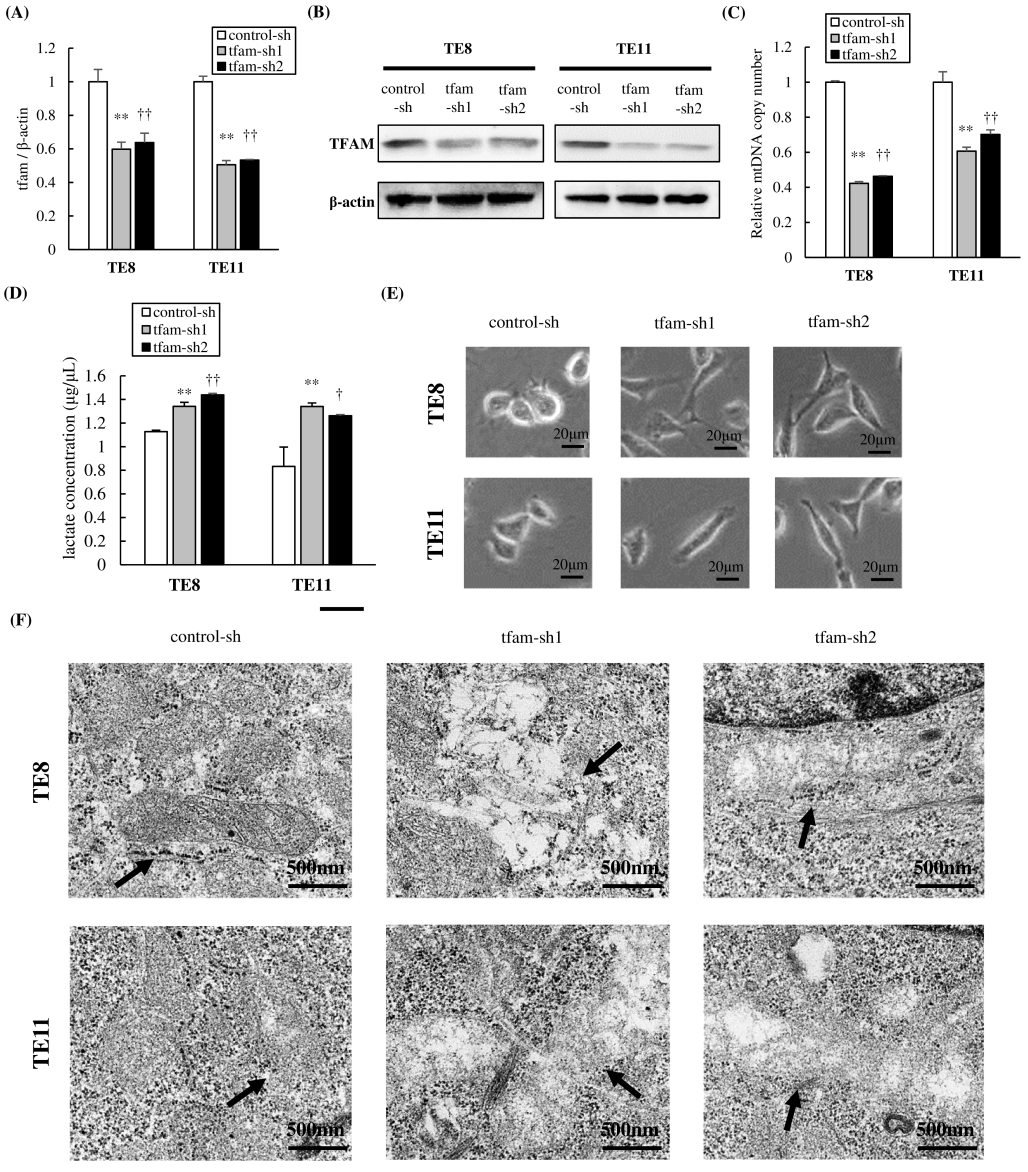
mtDNA-depleted TE8 and TE11 cells by knockdown of TFAM gene expression. **(A)** By short hairpin RNA knockdown of TFAM gene expression, the mRNA expression of TFAM was silenced to about 50% compared with non-target control cells in both TE8 and TE11 cells. **(B)** Immunoblotting showed downregulation of TFAM expression in tfam-sh cells. **(C)** Silencing TFAM reduced mtDNA copy number to about 40% in TE8 and about 60–70% in TE11 compared with non-target control cells. **(D)** The extracellular lactate concentrations of mtDNA-depleted cells were higher than control cells under normoxia (TE8: 1.34±0.03 or 1.44±0.01 vs 1.13±0.01 μg/μL, p<0.01; TE11: 1.34±0.03 (p<0.01) or 1.26±0.01 (p = 0.011) vs 0.83±0.16). **(E)** The TFAM knockdown cells showed spindle cell transformation. **(F)** The TFAM knockdown cells showed mitochondrial swelling and dissolved cristae compared with the control cells.

Downregulation of TFAM mRNA and protein levels was confirmed by qRT-PCR and immunoblotting, respectively (Fig 2A and 2B). As determined by qRT-PCR, the mRNA expression of TFAM was silenced to about 50% compared with non-target control cells in both TE8 and TE11 cells. The mtDNA copy numbers of TFAM knockdown cells were about 40% of the control cells in TE8, about 60–70% in TE11, and significantly lower than control cells (p<0.01; Fig 2C). To confirm the increased dependence on glycolysis due to mtDNA depletion, the lactate concentration of each cell was measured [34]. The extracellular lactate concentrations of mtDNA-depleted cells were higher than control cells (TE8: 1.34±0.03 or 1.44±0.01 vs 1.13±0.01 μg/μL, p<0.01; TE11: 1.34±0.03 (p<0.01) or 1.26±0.01 (p = 0.011) vs 0.83±0.16; Fig 2D).

The changes in morphology in TFAM knockdown cells were observed by optical microscopy and transmission electron microscopy. Compared with the control cells, the TFAM knockdown cells showed spindle cell transformation (Fig 2E) and mitochondrial swelling and dissolved cristae (bold arrow, Fig 2F).

We confirmed establishment of mtDNA-depleted cells by genetic suppression. Furthermore, we pharmacologically generated mtDNA-depleted cells by treatment with EtBr. The mtDNA copy number of cells treated by EtBr was 47.3±5.7% of the control cell in TE8, about 62.8±2.7% in TE11, and significantly lower than parental cells (p<0.01; S1A Fig).

### mtDNA-depleted ESCC cells have tolerance to hypoxia and acquire mesenchymal characteristics and stemness

**Fig 3:**
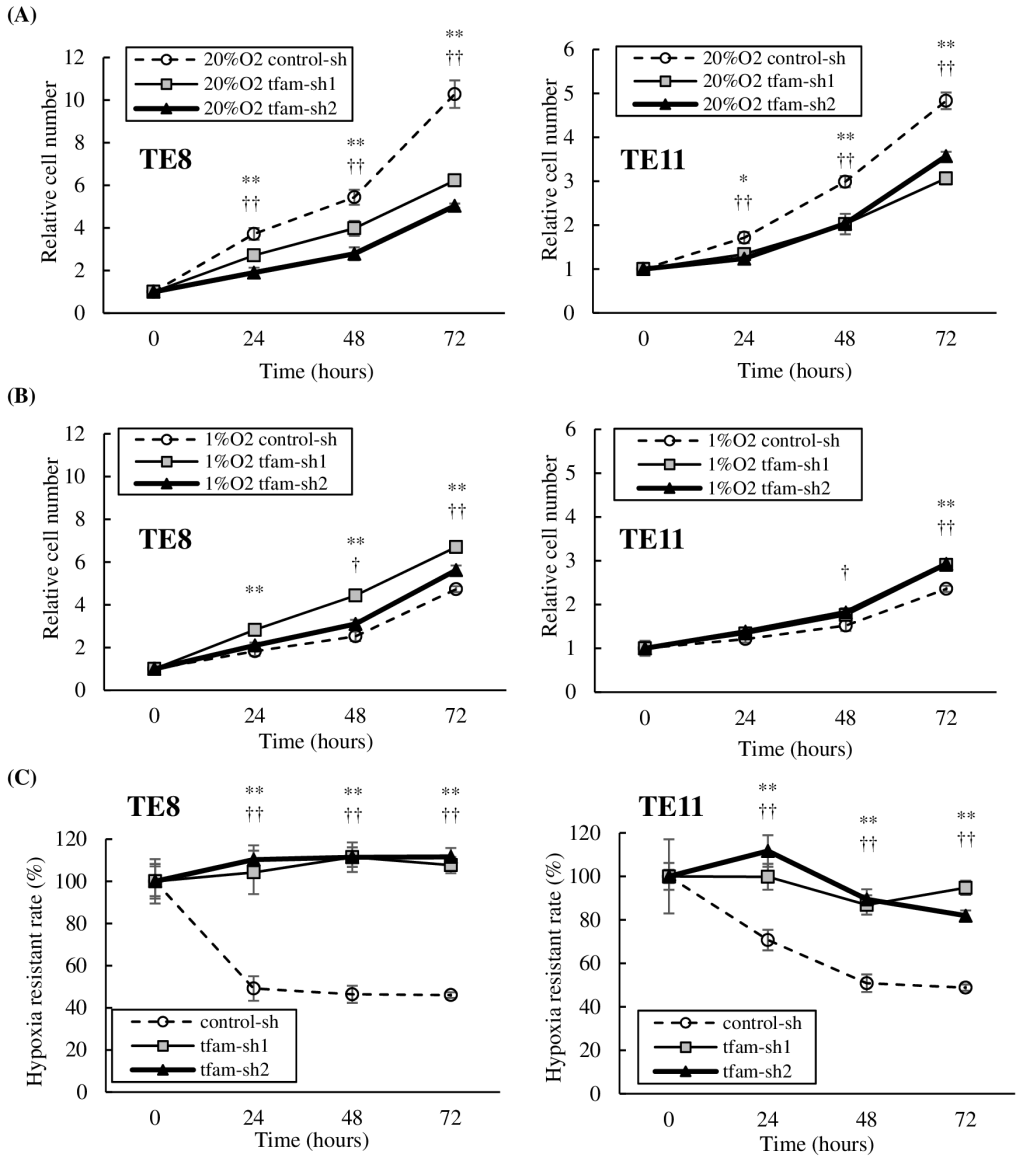
Cell proliferation and mRNA expression of mtDNA-depleted ESCC. **(A)** Cell proliferation of mtDNA-depleted cells and control cells were investigated under normoxia (20% O_2_) and hypoxia (1% O_2_). Under normoxia, the proliferation rates of mtDNA-depleted cells were significantly lower than control cells at 24 (TE8 tfam-sh1 or tfam-sh2 vs control-sh, p<0.01; TE11 tfam-sh1 vs control-sh, p = 0.011; TE11 tfam-sh2 vs control-sh, p<0.01), 48 (p<0.01), and 72 h (p<0.01). **(B)** Under hypoxia, the proliferation rate of control cells was decreased, but mtDNA-depleted cells proliferated at almost the same rate as under normoxia. **(C)** Hypoxia-resistant rates in mtDNA-depleted cells, as assessed by the ratio of the proliferation rate under hypoxia to that under normoxia, were significantly higher at 24, 48, and 72 h (p<0.01).

Cell proliferation of mtDNA-depleted cells and control cells was investigated under normoxia (20% O_2_) and hypoxia (1% O_2_). Both TE8 and TE11 cells with reduced mtDNA content proliferated slowly under normoxia (Fig 3A), but proliferated at almost the same rate even under hypoxia (Fig 3B and 3C). mtDNA depletion of TE8 and TE11 by treatment with EtBr also led to slow proliferation and tolerance to hypoxia, compared with parental cells (S1C and S1D Fig).

**Fig 4:**
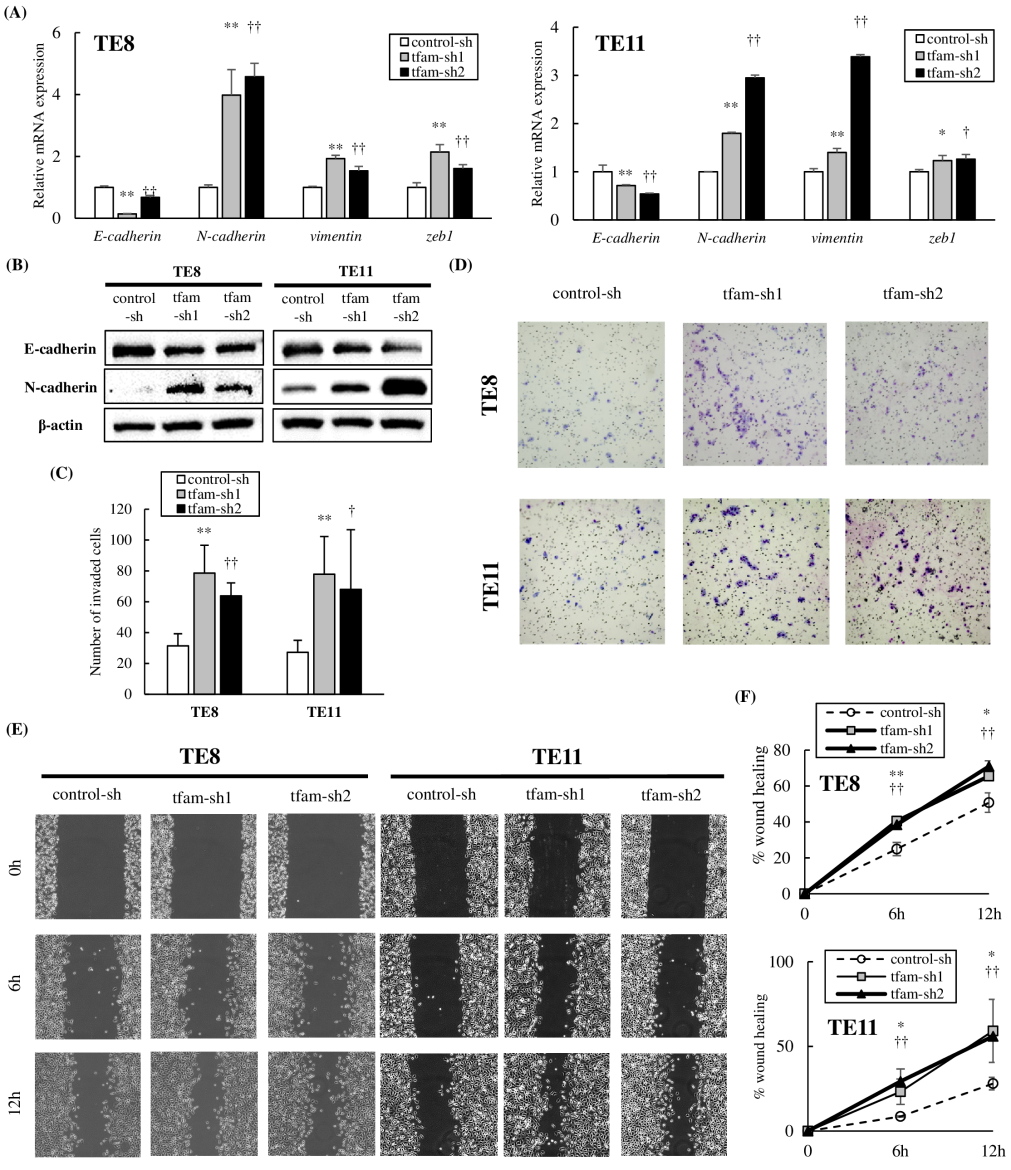
Acquired invasive and migratory potential in mtDNA-depleted ESCC. **(A)** The mRNA expression levels of four genes related to the epithelial–mesenchymal transition were analyzed by qPCR. Compared with control cells, in both TE8 and TE11 cells, *E-cadherin* expression in mtDNA-depleted cells was significantly decreased, while *N-cadherin*, *vimentin*, and *zeb-1* expression in mtDNA-depleted cells was significantly increased. **(B)** The protein levels of E-cadherin and N-cadherin were analyzed by immunoblotting. Compared with control cells, in both TE8 and TE11 cells, E-cadherin protein level in mtDNA-depleted cells was decreased, while N-cadherin protein level in mtDNA-depleted cells was increased. **(C, D)** Matrigel invasion assay was performed, and invasive cells at the bottom of the membrane were fixed and stained with the Diff-Quick stain kit. Both TE8 and TE11 mtDNA-depleted cells were significantly more invasive than control cells (TE8: 78.6±18.0 or 63.8±8.4 vs 31.4±7.8, p<0.01; TE11 tfam-sh1 vs control-sh: 77.8±24.5 vs 27.2±7.8, p<0.01; TE11 tfam-sh2 vs control-sh: 68.0±38.7 vs 27.2±7.8, p = 0.0495). **(E, F)** A confluent monolayer of cells was scratched using a pipette tip, and the wounded area was measured at three time points (0, 6, and 12 h). In both TE8 and TE11 cells, the wounded area was significantly decreased in mtDNA-depleted cells at 6 and 12 h compared with control cells (6h-TE8: 40.4±2.1 or 38.5±1.9 vs 24.9±3.7%, p<0.01; TE11 tfam-sh1 vs control-sh: 23.3±7.5 vs 8.6±0.4%, p = 0.028; TE11 tfam-sh2 vs control-sh: 29.4±7.3 vs 8.6±0.4%, p<0.01; 12h-TE8 tfam-sh1 vs control-sh: 65.6±1.6 vs 50.8±5.4%, p = 0.011;12h-TE8 tfam-sh2 vs control-sh: 71.0±3.1 vs 50.8±5.4%, p<0.01; TE11 tfam-sh1 vs control-sh: 59.2±18.6 vs 28.0±3.7%, p = 0.047; TE11 tfam-sh2 vs control-sh: 56.0±1.5 vs 28.0±3.7%, p<0.01).

To investigate the biology of mtDNA-depleted cells, we assessed the gene expression associated with epithelial–mesenchymal transition (EMT), which was considered to play an important role in tumor progression [35–37]. In mtDNA-depleted TE8 and TE11, the mRNA expression of *E-cadherin* was downregulated, whereas the mRNA expression of *N-cadherin*, *vimentin*, and *zeb1* was upregulated (Fig 4A). Immunoblotting analysis also showed downregulated E-cadherin and upregulated N-cadherin protein in mtDNA-depleted TE8 and TE11 (Fig 4B). Based on this result, we consequently assessed cell migration and invasion. Matrigel invasion assay and the scratch-wound healing migration assay showed that mtDNA-depleted TE8 and TE11 were significantly more invasive and migrated more than control-sh cells (Fig 4C–4F). The mtDNA-depleted cells generated by treatment with EtBr were also more invasive and migratory than parental cells and had the same changes in the four genes and two proteins related to EMT (S2 Fig).

**Fig 5:**
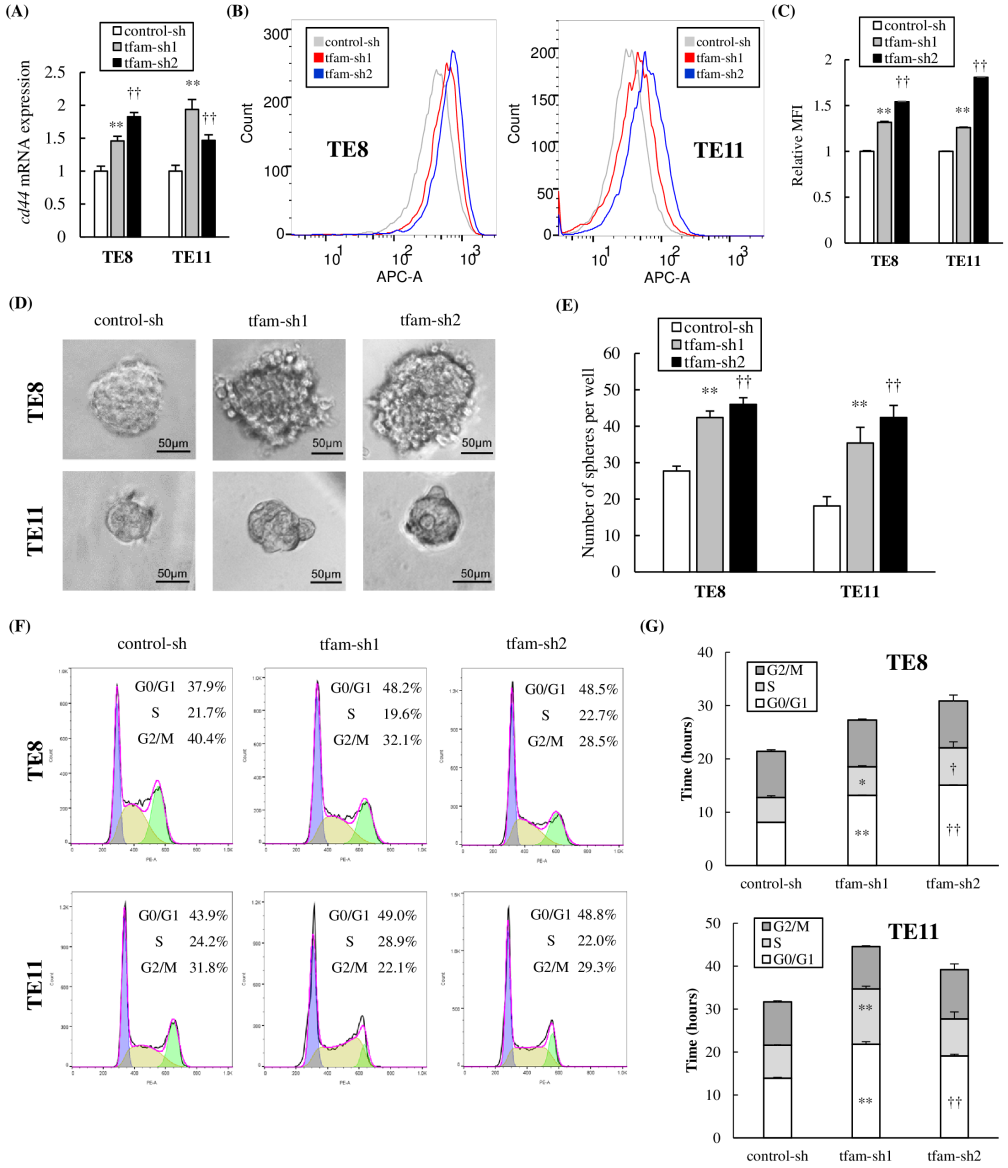
mtDNA-depleted cells have stem-like characteristics. **(A)** The mRNA expression level of *cd44* was analyzed by qPCR. In both TE8 and TE11 cells, *cd44* expression of mtDNA-depleted cells was significantly increased compared with control cells. **(B, C)** The protein expressions of CD44 were analyzed by flow cytometry using APC-CD44. MtDNA-depleted cells had higher protein expression of CD44 than control cells. **(D)** Spheres formed by both TE8 and TE11 cells. **(E)** mtDNA-depleted cells formed significantly more spheres than control cells (TE8: 42.4±1.8 or 46.0±1.8 vs 27.7±1.3; TE11: 35.4±4.3 or 42.4±3.3 vs 18.1±2.6; p<0.01). **(F, G)** mtDNA-depleted cells and control cells were stained with propidium iodide and analyzed by flow cytometry. The duration in G0/G1 phase was significantly longer in mtDNA-depleted cells than in control cells (TE8: 13.2±0.1 or 15.1±0.1 vs 8.1±0.0 h; TE11: 21.8±0.6 or 19.1±0.4 vs 13.9±0.2 h; p<0.01).

The mtDNA-depleted ESCC cells also had high mRNA and protein expression of *cd44*, which is considered a cancer stem cell marker in ESCC (Fig 5A–5C) [38–40]. The tumor sphere formation assay showed that mtDNA-depleted cells formed significantly more spheres than control cells (TE8: 42.4±1.8 or 46.0±1.8 vs 27.7±1.3; TE11: 35.4±4.3 or 42.4±3.3 vs 18.1±2.6; p<0.01) (Fig 5D and 5E). Moreover, the cell cycle assay revealed that the duration in G0/G1 phase was significantly longer in mtDNA-depleted cells than in control cells (TE8: 13.2±0.1 or 15.1±0.1 vs 8.1±0.0 h; TE11: 21.8±0.6 or 19.1±0.4 vs 13.9±0.2 h; p<0.01) (Fig 5F and 5G). In mtDNA-depleted cells generated by EtBr treatment, the same changes were also induced (S3 Fig). These results suggested that mtDNA-depleted ESCC cells acquired mesenchymal characteristics and stemness.

## Discussion

The origin of the mitochondrion is widely considered to have been a bacterial progenitor incorporated via symbiosis within an eukaryotic host cell [41, 42]. There is a hypothesis that cancer is in an atavistic condition [43, 44]. Hence, we hypothesized that the cancer may return to a state more like a cell without mitochondria. Mitochondrial defects in cancers have been studied before [45–48], but few reports have addressed them in esophageal cancer. Two recent reports investigated the mutation of mtDNA in esophageal cancer and revealed that D-loop alterations were frequent and that the alteration was associated with poor prognosis [49, 50]. The mutation in the D-loop region also has been linked to reduced mtDNA copy number in other cancers [51, 52].

We focused on the correlation between mtDNA copy number and clinicopathological features in ESCC. In this report, we investigated 100 clinical samples, including non-cancerous tissues, from 80 ESCC patients. We found that mtDNA copy number in ESCC was significantly lower than that of non-cancerous tissues and that low mtDNA copy number was associated with tumor progression and poor prognosis. Alterations in mtDNA copy numbers in other human cancers have been previously studied. In gastric cancer, the mtDNA copy number in cancerous tissues was significantly lower than in the corresponding non-cancerous tissues, and the quantitative changes in mtDNA demonstrated a significant decrease particularly in advanced cases [24]. In colorectal cancer, the mtDNA copy number in cancerous tissue was lower than the corresponding non-cancerous tissue and correlated with poor prognosis [21]. The results of our study with ESCC were consistent with these observations in other cancers. The mtDNA copy number of a tumor may be decreased and low mtDNA copy number may be associated with tumor progression. Some reports showed that mtDNA copy number of ESCC was increased, but the change might be a compensation for the damaged mtDNA to maintain mitochondrial function as the author described [53–56].

Hypoxia is a characteristic feature of locally advanced solid tumors [57, 58]. Our results showed that mtDNA-depleted ESCC cells had tolerance to hypoxia with activating glycolysis and that the mtDNA copy number of cancerous tissue was negatively correlated with depth of tumor invasion. Hence, we hypothesized that the mtDNA copy number of advanced tumors was decreased as a result of adaptation to hypoxia. The mechanism of this adaptation warrants further study.

We also found that ESCC cells with low mtDNA copy number have mesenchymal characteristics and cancer stemness, which play an important role in cancer progression [35–37, 57, 59]. Cells with low mtDNA copy number have higher migration activity in renal cell carcinoma [30] and a stem-like, migratory, and invasive phenotype in breast cancer [27]. The results of our *in vitro* assay were consistent with those for other cancers and our findings in terms of tumor progression.

Moreover, in this study, transmission electron microscopy revealed that TFAM knockdown cells showed mitochondrial swelling and dissolved cristae. Although there is no report on the ultrastructure of TFAM knockdown cells, the change in morphology is similar to that reported in drug-resistant tumor cells [60, 61]. Hence, mtDNA-depleted ESCC cells may possibly have resistance to anticancer drugs.

The limitation of this study was that it did not clarify how cancer decreases mtDNA copy number or how low mtDNA copy number induces EMT and stemness. The underlying mechanism, the agents for upregulating mtDNA copy number, and the correlation between drug resistance and mtDNA copy number warrant further investigation.

## Conclusions

The present study showed that low mtDNA copy number is correlated with tumor invasion, epithelial-to-mesenchymal transition, cancer stemness, and poor prognosis in ESCC.

## Supporting information

S1 Fig mtDNA-depleted TE8 and TE11 cells by treatment with EtBr. **(A)** Treatment with EtBr reduced mtDNA copy number to 47.3% in TE8 and 62.8% in TE11 cells compared with parental cells. **(B)** The EtBr treated cells showed spindle cell transformation. **(C)** Under normoxia, the proliferation rates of mtDNA-depleted cells by EtBr were significantly lower than in parental cells at 24 (TE8, p = 0.042; TE11, p<0.01), 48(TE8, p<0.01; TE11, p = 0.030), and 72 h (p<0.01). **(D)** Under hypoxia, the proliferation rate of parental cells was decreased, but mtDNA-depleted cells generated by EtBr exposure proliferated at almost the same rate.
(TIF)

S2 Fig Invasive and migratory potential of mtDNA-depleted cells by treatment with EtBr. **(A)** The mRNA levels of four genes related to epithelial–mesenchymal transition were analyzed by qPCR. In both TE8 and TE11 cells, *E-cadherin* expression in mtDNA-depleted cells was significantly decreased, while *N-cadherin*, *vimentin*, and *zeb-1* expression in mtDNA-depleted cells was significantly increased, compared with parental cells. **(B)** The protein levels of E-cadherin and N-cadherin were analyzed by immunoblotting. Compared with parental cells, in both TE8 and TE11 cells, E-cadherin protein level in mtDNA-depleted cells was decreased, while N-cadherin protein level in mtDNA-depleted cells was increased. **(C, D)** Both TE8 and TE11 mtDNA-depleted cells were significantly more invasive than parental cells (TE8: 64.3±10.0 vs 25.3±3.5; TE11: 126.0±21.4 vs 52.7±15.5, p<0.01). **(E, F)** The confluent monolayer of cells was scratched using a pipette tip, and the wounded area was measured at two time points (0 and 12 h). In both TE8 and TE11 cells, the wounded area was significantly decreased in mtDNA-depleted cells at 12 h, compared with parental cells (TE8: 66.0±6.0 vs 51.1±3.4%, p = 0.038; TE11: 40.6±3.2 vs 31.6±4.1%; p = 0.041).
(TIF)

S3 Fig mtDNA-depleted cells by treatment with EtBr also have stem-like characteristics. **(A)** In both TE8 and TE11 cells, *cd44* expression of mtDNA-depleted cells was significantly increased compared with parental cells. **(B, C)** The protein expressions of CD44 were analyzed by flow cytometry using APC-CD44. MtDNA-depleted cells by EtBr treatment had higher protein expression of CD44 than parental cells. **(D)** Spheres formed by both TE8 and TE11 cells. **(E)** mtDNA-depleted cells formed significantly more spheres than parental cells (61.8±1.7 vs 46.7±2.0; TE11: 60.6±6.0 vs 48.3±2.3; p<0.01) **(F, G)** The duration in G0/G1 phase was significantly longer in mtDNA-depleted cells than in parental cells (TE8: 17.0±0.2 vs 7.9±0.1 h; TE11: 34.9±0.7 vs 15.0±0.2 h; p<0.01).
(TIF)

S1 Table Prognostic analysis regarding overall survival. (XLSX)

## References

[1] Ferlay J, Soerjomataram I, Dikshit R, Eser S, Mathers C, Rebelo M, et al Cancer incidence and mortality worldwide: sources, methods and major patterns in GLOBOCAN 2012. International journal of cancer. 2015;136(5):E359–86. doi: 10.1002/ijc.29210 . 25220842 10.1002/ijc.29210

[2] Pennathur A, Gibson MK, Jobe BA, Luketich JD. Oesophageal carcinoma. Lancet. 2013;381(9864):400–12. doi: 10.1016/S0140-6736(12)60643-6 . 23374478 10.1016/S0140-6736(12)60643-6

[3] Siegel RL, Miller KD, Jemal A. Cancer Statistics, 2017. CA: a cancer journal for clinicians. 2017;67(1):7–30. doi: 10.3322/caac.21387 . 28055103 10.3322/caac.21387

[4] Torre LA, Bray F, Siegel RL, Ferlay J, Lortet-Tieulent J, Jemal A. Global cancer statistics, 2012. CA: a cancer journal for clinicians. 2015;65(2):87–108. doi: 10.3322/caac.21262 . 25651787 10.3322/caac.21262

[5] Ando N, Kato H, Igaki H, Shinoda M, Ozawa S, Shimizu H, et al A randomized trial comparing postoperative adjuvant chemotherapy with cisplatin and 5-fluorouracil versus preoperative chemotherapy for localized advanced squamous cell carcinoma of the thoracic esophagus (JCOG9907). Annals of surgical oncology. 2012;19(1):68–74. doi: 10.1245/s10434-011-2049-9 . 21879261 10.1245/s10434-011-2049-9

[6] Colvin H. Gastroenterological surgery in Japan: The past, the present and the future. Ann Gastroenterol Surg. 2017;1(1):5–10. 10.1002/ags3.12008 PMC5881296 29863129

[7] Sjoquist KM, Burmeister BH, Smithers BM, Zalcberg JR, Simes RJ, Barbour A, et al Survival after neoadjuvant chemotherapy or chemoradiotherapy for resectable oesophageal carcinoma: an updated meta-analysis. The Lancet Oncology. 2011;12(7):681–92. doi: 10.1016/S1470-2045(11)70142-5 . 21684205 10.1016/S1470-2045(11)70142-5

[8] van Hagen P, Hulshof MC, van Lanschot JJ, Steyerberg EW, van Berge Henegouwen MI, Wijnhoven BP, et al Preoperative chemoradiotherapy for esophageal or junctional cancer. The New England journal of medicine. 2012;366(22):2074–84. doi: 10.1056/NEJMoa1112088 . 22646630 10.1056/NEJMoa1112088

[9] Miyata H, Yoshioka A, Yamasaki M, Nushijima Y, Takiguchi S, Fujiwara Y, et al Tumor budding in tumor invasive front predicts prognosis and survival of patients with esophageal squamous cell carcinomas receiving neoadjuvant chemotherapy. Cancer. 2009;115(14):3324–34. doi: 10.1002/cncr.24390 . 19452547 10.1002/cncr.24390

[10] Tachimori Y, Ozawa S, Numasaki H, Ishihara R, Matsubara H, Muro K, et al Comprehensive Registry of Esophageal Cancer in Japan, 2010. Esophagus: official journal of the Japan Esophageal Society. 2017;14(3):189–214. doi: 10.1007/s10388-017-0578-4 ; PubMed Central PMCID: PMC5486463. 28725168 10.1007/s10388-017-0578-4 PMC5486463

[11] Attardi G, Schatz G. Biogenesis of mitochondria. Annual review of cell biology. 1988;4:289–333. doi: 10.1146/annurev.cb.04.110188.001445 . 2461720 10.1146/annurev.cb.04.110188.001445

[12] Lee HC, Wei YH. Mitochondrial role in life and death of the cell. Journal of biomedical science. 2000;7(1):2–15. doi: 25424 . 10644884 10.1007/BF02255913

[13] Newmeyer DD, Ferguson-Miller S. Mitochondria: releasing power for life and unleashing the machineries of death. Cell. 2003;112(4):481–90. . 12600312 10.1016/s0092-8674(03)00116-8

[14] Bogenhagen DF. Mitochondrial DNA nucleoid structure. Biochimica et biophysica acta. 2012;1819(9–10):914–20. doi: 10.1016/j.bbagrm.2011.11.005 . 22142616 10.1016/j.bbagrm.2011.11.005

[15] Chen XJ, Butow RA. The organization and inheritance of the mitochondrial genome. Nature reviews Genetics. 2005;6(11):815–25. doi: 10.1038/nrg1708 . 16304597 10.1038/nrg1708

[16] Lee HC, Wei YH. Mitochondrial biogenesis and mitochondrial DNA maintenance of mammalian cells under oxidative stress. The international journal of biochemistry & cell biology. 2005;37(4):822–34. doi: 10.1016/j.biocel.2004.09.010 . 15694841 10.1016/j.biocel.2004.09.010

[17] Kukat C, Wurm CA, Spahr H, Falkenberg M, Larsson NG, Jakobs S. Super-resolution microscopy reveals that mammalian mitochondrial nucleoids have a uniform size and frequently contain a single copy of mtDNA. Proceedings of the National Academy of Sciences of the United States of America. 2011;108(33):13534–9. doi: 10.1073/pnas.1109263108 ; PubMed Central PMCID: PMC3158146. 21808029 10.1073/pnas.1109263108 PMC3158146

[18] Robin ED, Wong R. Mitochondrial DNA molecules and virtual number of mitochondria per cell in mammalian cells. Journal of cellular physiology. 1988;136(3):507–13. doi: 10.1002/jcp.1041360316 . 3170646 10.1002/jcp.1041360316

[19] Kim MM, Clinger JD, Masayesva BG, Ha PK, Zahurak ML, Westra WH, et al Mitochondrial DNA quantity increases with histopathologic grade in premalignant and malignant head and neck lesions. Clinical cancer research: an official journal of the American Association for Cancer Research. 2004;10(24):8512–5. doi: 10.1158/1078-0432.CCR-04-0734 . 15623632 10.1158/1078-0432.CCR-04-0734

[20] Yu M, Zhou Y, Shi Y, Ning L, Yang Y, Wei X, et al Reduced mitochondrial DNA copy number is correlated with tumor progression and prognosis in Chinese breast cancer patients. IUBMB life. 2007;59(7):450–7. doi: 10.1080/15216540701509955 . 17654121 10.1080/15216540701509955

[21] Cui H, Huang P, Wang Z, Zhang Y, Zhang Z, Xu W, et al Association of decreased mitochondrial DNA content with the progression of colorectal cancer. BMC cancer. 2013;13:110 doi: 10.1186/1471-2407-13-110 ; PubMed Central PMCID: PMC3606376. 23497023 10.1186/1471-2407-13-110 PMC3606376

[22] Zhang Y, Qu Y, Gao K, Yang Q, Shi B, Hou P, et al High copy number of mitochondrial DNA (mtDNA) predicts good prognosis in glioma patients. American journal of cancer research. 2015;5(3):1207–16. ; PubMed Central PMCID: PMC4449448. 26045999 PMC4449448

[23] Wu CW, Yin PH, Hung WY, Li AF, Li SH, Chi CW, et al Mitochondrial DNA mutations and mitochondrial DNA depletion in gastric cancer. Genes, chromosomes & cancer. 2005;44(1):19–28. doi: 10.1002/gcc.20213 . 15892105 10.1002/gcc.20213

[24] Wen SL, Zhang F, Feng S. Decreased copy number of mitochondrial DNA: A potential diagnostic criterion for gastric cancer. Oncology letters. 2013;6(4):1098–102. doi: 10.3892/ol.2013.1492 ; PubMed Central PMCID: PMC3796381. 24137470 10.3892/ol.2013.1492 PMC3796381

[25] van Osch FH, Voets AM, Schouten LJ, Gottschalk RW, Simons CC, van Engeland M, et al Mitochondrial DNA copy number in colorectal cancer: between tissue comparisons, clinicopathological characteristics and survival. Carcinogenesis. 2015;36(12):1502–10. doi: 10.1093/carcin/bgv151 . 26476438 10.1093/carcin/bgv151

[26] Rice TW, Patil DT, Blackstone EH. 8th edition AJCC/UICC staging of cancers of the esophagus and esophagogastric junction: application to clinical practice. Annals of cardiothoracic surgery. 2017;6(2):119–30. doi: 10.21037/acs.2017.03.14 ; PubMed Central PMCID: PMC5387145. 28447000 10.21037/acs.2017.03.14 PMC5387145

[27] Guha M, Srinivasan S, Ruthel G, Kashina AK, Carstens RP, Mendoza A, et al Mitochondrial retrograde signaling induces epithelial-mesenchymal transition and generates breast cancer stem cells. Oncogene. 2014;33(45):5238–50. doi: 10.1038/onc.2013.467 ; PubMed Central PMCID: PMC4921233. 24186204 10.1038/onc.2013.467 PMC4921233

[28] King MP, Attardi G. Human cells lacking mtDNA: repopulation with exogenous mitochondria by complementation. Science. 1989;246(4929):500–3. . 2814477 10.1126/science.2814477

[29] Lee W, Choi HI, Kim MJ, Park SY. Depletion of mitochondrial DNA up-regulates the expression of MDR1 gene via an increase in mRNA stability. Experimental & molecular medicine. 2008;40(1):109–17. doi: 10.3858/emm.2008.40.1.109 ; PubMed Central PMCID: PMC2679319. 18305404 10.3858/emm.2008.40.1.109 PMC2679319

[30] Lin CS, Lee HT, Lee MH, Pan SC, Ke CY, Chiu AW, et al Role of Mitochondrial DNA Copy Number Alteration in Human Renal Cell Carcinoma. International journal of molecular sciences. 2016;17(6). doi: 10.3390/ijms17060814 ; PubMed Central PMCID: PMC4926348. 27231905 10.3390/ijms17060814 PMC4926348

[31] Campbell CT, Kolesar JE, Kaufman BA. Mitochondrial transcription factor A regulates mitochondrial transcription initiation, DNA packaging, and genome copy number. Biochimica et biophysica acta. 2012;1819(9–10):921–9. doi: 10.1016/j.bbagrm.2012.03.002 . 22465614 10.1016/j.bbagrm.2012.03.002

[32] Schneider CA, Rasband WS, Eliceiri KW. NIH Image to ImageJ: 25 years of image analysis. Nature methods. 2012;9(7):671–5. ; PubMed Central PMCID: PMC5554542. 22930834 10.1038/nmeth.2089 PMC5554542

[33] Rasband WS. ImageJ, U. S. National Institutes of Health, Bethesda, Maryland, USA, https://imagej.nih.gov/ij/. 1997–2016.

[34] Yu M, Shi Y, Wei X, Yang Y, Zang F, Niu R. Mitochondrial DNA depletion promotes impaired oxidative status and adaptive resistance to apoptosis in T47D breast cancer cells. European journal of cancer prevention: the official journal of the European Cancer Prevention Organisation. 2009;18(6):445–57. doi: 10.1097/CEJ.0b013e32832f9bd6 . 19609211 10.1097/CEJ.0b013e32832f9bd6

[35] Usami Y, Satake S, Nakayama F, Matsumoto M, Ohnuma K, Komori T, et al Snail-associated epithelial-mesenchymal transition promotes oesophageal squamous cell carcinoma motility and progression. The Journal of pathology. 2008;215(3):330–9. doi: 10.1002/path.2365 . 18491351 10.1002/path.2365

[36] Zhou P, Li B, Liu F, Zhang M, Wang Q, Liu Y, et al The epithelial to mesenchymal transition (EMT) and cancer stem cells: implication for treatment resistance in pancreatic cancer. Molecular cancer. 2017;16(1):52 doi: 10.1186/s12943-017-0624-9 ; PubMed Central PMCID: PMC5331747. 28245823 10.1186/s12943-017-0624-9 PMC5331747

[37] Wen J, Luo KJ, Liu QW, Wang G, Zhang MF, Xie XY, et al The epithelial-mesenchymal transition phenotype of metastatic lymph nodes impacts the prognosis of esophageal squamous cell carcinoma patients. Oncotarget. 2016;7(25):37581–8. doi: 10.18632/oncotarget.9036 ; PubMed Central PMCID: PMC5122333. 27147562 10.18632/oncotarget.9036 PMC5122333

[38] Qian X, Tan C, Wang F, Yang B, Ge Y, Guan Z, et al Esophageal cancer stem cells and implications for future therapeutics. OncoTargets and therapy. 2016;9:2247–54. doi: 10.2147/OTT.S103179 ; PubMed Central PMCID: PMC4846051. 27143920 10.2147/OTT.S103179 PMC4846051

[39] Rassouli FB, Matin MM, Bahrami AR, Ghaffarzadegan K, Cheshomi H, Lari S, et al Evaluating stem and cancerous biomarkers in CD15+CD44+ KYSE30 cells. Tumour biology: the journal of the International Society for Oncodevelopmental Biology and Medicine. 2013;34(5):2909–20. doi: 10.1007/s13277-013-0853-5 . 23797812 10.1007/s13277-013-0853-5

[40] Wang D, Plukker JTM, Coppes RP. Cancer stem cells with increased metastatic potential as a therapeutic target for esophageal cancer. Seminars in cancer biology. 2017;44:60–6. doi: 10.1016/j.semcancer.2017.03.010 . 28366541 10.1016/j.semcancer.2017.03.010

[41] Gray MW. Rickettsia, typhus and the mitochondrial connection. Nature. 1998;396(6707):109–10. doi: 10.1038/24030 . 9823885 10.1038/24030

[42] Gray MW, Burger G, Lang BF. Mitochondrial evolution. Science. 1999;283(5407):1476–81. . 10066161 10.1126/science.283.5407.1476

[43] Davies PC, Lineweaver CH. Cancer tumors as Metazoa 1.0: tapping genes of ancient ancestors. Physical biology. 2011;8(1):015001 doi: 10.1088/1478-3975/8/1/015001 ; PubMed Central PMCID: PMC3148211. 21301065 10.1088/1478-3975/8/1/015001 PMC3148211

[44] Thomas F, Ujvari B, Renaud F, Vincent M. Cancer adaptations: Atavism, de novo selection, or something in between? BioEssays: news and reviews in molecular, cellular and developmental biology. 2017;39(8). doi: 10.1002/bies.201700039 . 28691339 10.1002/bies.201700039

[45] Boland ML, Chourasia AH, Macleod KF. Mitochondrial dysfunction in cancer. Frontiers in oncology. 2013;3:292 doi: 10.3389/fonc.2013.00292 ; PubMed Central PMCID: PMC3844930. 24350057 10.3389/fonc.2013.00292 PMC3844930

[46] Carew JS, Huang P. Mitochondrial defects in cancer. Molecular cancer. 2002;1:9 doi: 10.1186/1476-4598-1-9 ; PubMed Central PMCID: PMC149412. 12513701 10.1186/1476-4598-1-9 PMC149412

[47] Tokarz P, Blasiak J. Role of mitochondria in carcinogenesis. Acta biochimica Polonica. 2014;61(4):671–8. . 25493442

[48] Yu M. Generation, function and diagnostic value of mitochondrial DNA copy number alterations in human cancers. Life sciences. 2011;89(3–4):65–71. doi: 10.1016/j.lfs.2011.05.010 . 21683715 10.1016/j.lfs.2011.05.010

[49] Tan DJ, Chang J, Liu LL, Bai RK, Wang YF, Yeh KT, et al Significance of somatic mutations and content alteration of mitochondrial DNA in esophageal cancer. BMC cancer. 2006;6:93 doi: 10.1186/1471-2407-6-93 ; PubMed Central PMCID: PMC1459869. 16620376 10.1186/1471-2407-6-93 PMC1459869

[50] Zhang R, Wang R, Zhang F, Wu C, Fan H, Li Y, et al Single nucleotide polymorphisms in the mitochondrial displacement loop and outcome of esophageal squamous cell carcinoma. Journal of experimental & clinical cancer research: CR. 2010;29:155 doi: 10.1186/1756-9966-29-155 ; PubMed Central PMCID: PMC3000395. 21110870 10.1186/1756-9966-29-155 PMC3000395

[51] Kabekkodu SP, Bhat S, Mascarenhas R, Mallya S, Bhat M, Pandey D, et al Mitochondrial DNA variation analysis in cervical cancer. Mitochondrion. 2014;16:73–82. doi: 10.1016/j.mito.2013.07.001 . 23851045 10.1016/j.mito.2013.07.001

[52] Lee HC, Li SH, Lin JC, Wu CC, Yeh DC, Wei YH. Somatic mutations in the D-loop and decrease in the copy number of mitochondrial DNA in human hepatocellular carcinoma. Mutation research. 2004;547(1–2):71–8. doi: 10.1016/j.mrfmmm.2003.12.011 . 15013701 10.1016/j.mrfmmm.2003.12.011

[53] Li H, Tian Z, Zhang Y, Yang Q, Shi B, Hou P, et al Increased copy number of mitochondrial DNA predicts poor prognosis of esophageal squamous cell carcinoma. Oncology letters. 2018;15:1014–20. 29422970 10.3892/ol.2017.7416 PMC5772934

[54] Lin CS, Chang SC, Wang LS, Chou TY, Hsu WH, Wu YC, et al The role of mitochondrial DNA alterations in esophageal squamous cell carcinomas. The Journal of thoracic and cardiovascular surgery. 2010;139(1):189–97 e4. doi: 10.1016/j.jtcvs.2009.04.007 . 19660406 10.1016/j.jtcvs.2009.04.007

[55] Lin CS, Lee HT, Lee SY, Shen YA, Wang LS, Chen YJ, et al High mitochondrial DNA copy number and bioenergetic function are associated with tumor invasion of esophageal squamous cell carcinoma cell lines. International journal of molecular sciences. 2012;13(9):11228–46. doi: 10.3390/ijms130911228 ; PubMed Central PMCID: PMC3472741. 23109849 10.3390/ijms130911228 PMC3472741

[56] Liu Z, Zhao Z, Zhao Q, Li S, Gao D, Pang X, et al Change and Significance of Mitochondrial DNA copy number in Esophageal Squamous Cell Carcinoma. Chinese Journal of Clinical Oncology. 2007;4(1):29–32.

[57] Harris AL. Hypoxia—a key regulatory factor in tumour growth. Nature reviews Cancer. 2002;2(1):38–47. doi: 10.1038/nrc704 . 11902584 10.1038/nrc704

[58] Vaupel P, Mayer A. Hypoxia in cancer: significance and impact on clinical outcome. Cancer metastasis reviews. 2007;26(2):225–39. doi: 10.1007/s10555-007-9055-1 . 17440684 10.1007/s10555-007-9055-1

[59] Okamoto K, Ninomiya I, Ohbatake Y, Hirose A, Tsukada T, Nakanuma S, et al Expression status of CD44 and CD133 as a prognostic marker in esophageal squamous cell carcinoma treated with neoadjuvant chemotherapy followed by radical esophagectomy. Oncology reports. 2016;36(6):3333–42. doi: 10.3892/or.2016.5133 . 27748881 10.3892/or.2016.5133

[60] Okon IS, Coughlan KA, Zhang M, Wang Q, Zou MH. Gefitinib-mediated reactive oxygen specie (ROS) instigates mitochondrial dysfunction and drug resistance in lung cancer cells. The Journal of biological chemistry. 2015;290(14):9101–10. doi: 10.1074/jbc.M114.631580 ; PubMed Central PMCID: PMC4423695. 25681445 10.1074/jbc.M114.631580 PMC4423695

[61] Saitou M, Isonishi S, Hamada T, Kiyokawa T, Tachibana T, Ishikawa H, et al Mitochondrial ultrastructure-associated chemotherapy response in ovarian cancer. Oncology reports. 2009;21(1):199–204. . 19082462

