# Supplementary figures and images for: Esophageal squamous cell carcinoma with low mitochondrial copy number has mesenchymal and stem-like characteristics, and contributes to poor prognosis
Source: PLoS One. 2018 Feb 15;13(2):e0193159. doi: 10.1371/journal.pone.0193159 (PMC5814088; doi:10.1371/journal.pone.0193159)

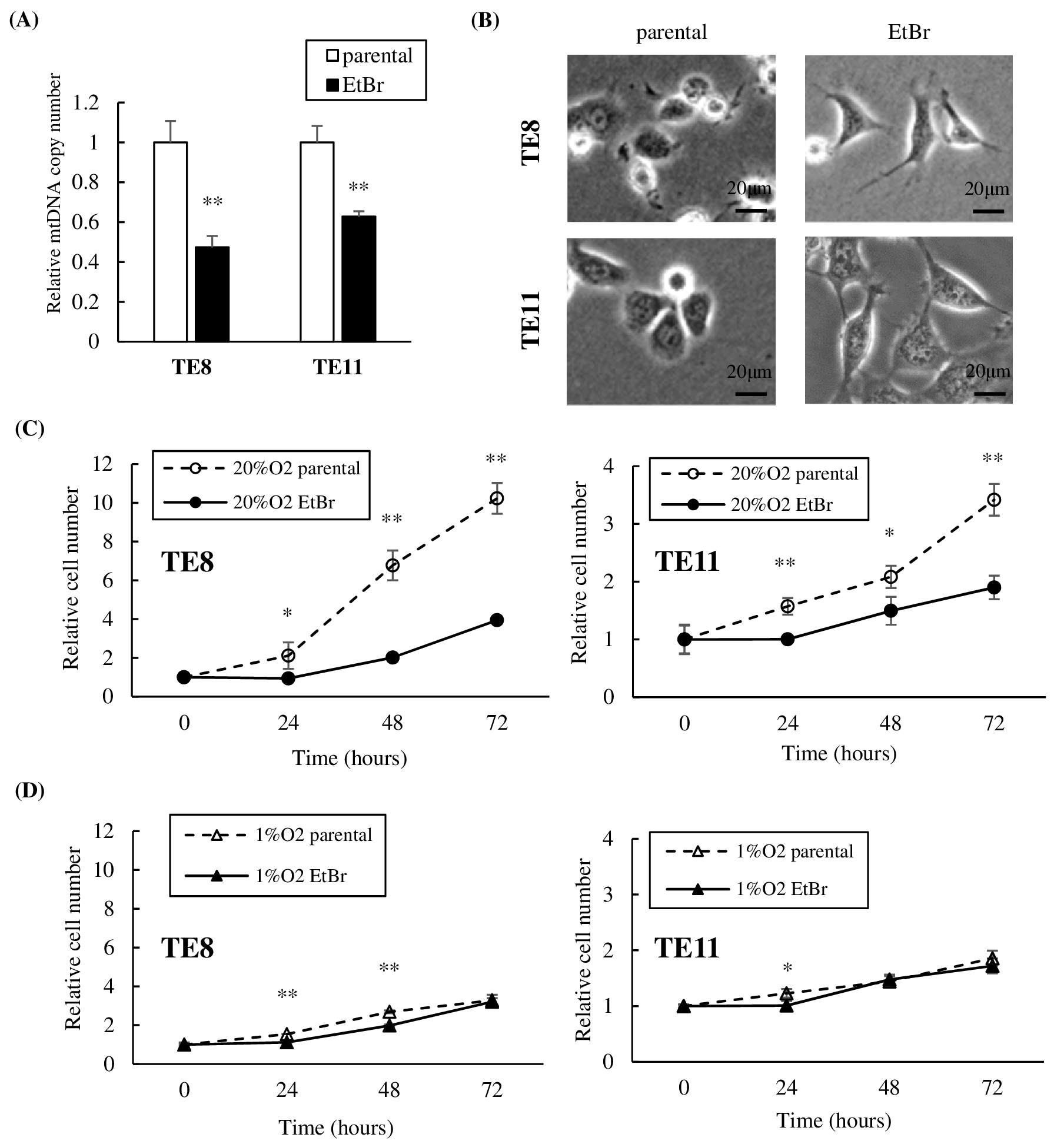

Supplement: S1 Fig — (A) Treatment with EtBr reduced mtDNA copy number to 47.3% in TE8 and 62.8% in TE11 cells compared with parental cells. (B) The EtBr treated cells showed spindle cell transformation. (C) Under normoxia, the proliferation rates of mtDNA-depleted cells by EtBr were significantly lower than in parental cells at 24 (TE8, p = 0.042; TE11, p<0.01), 48(TE8, p<0.01; TE11, p = 0.030), and 72 h (p<0.01). (D) Under hypoxia, the proliferation rate of parental cells was decreased, but mtDNA-depleted cells generated by EtBr exposure proliferated at almost the same rate. (TIF) [file pone.0193159.s001.tif]

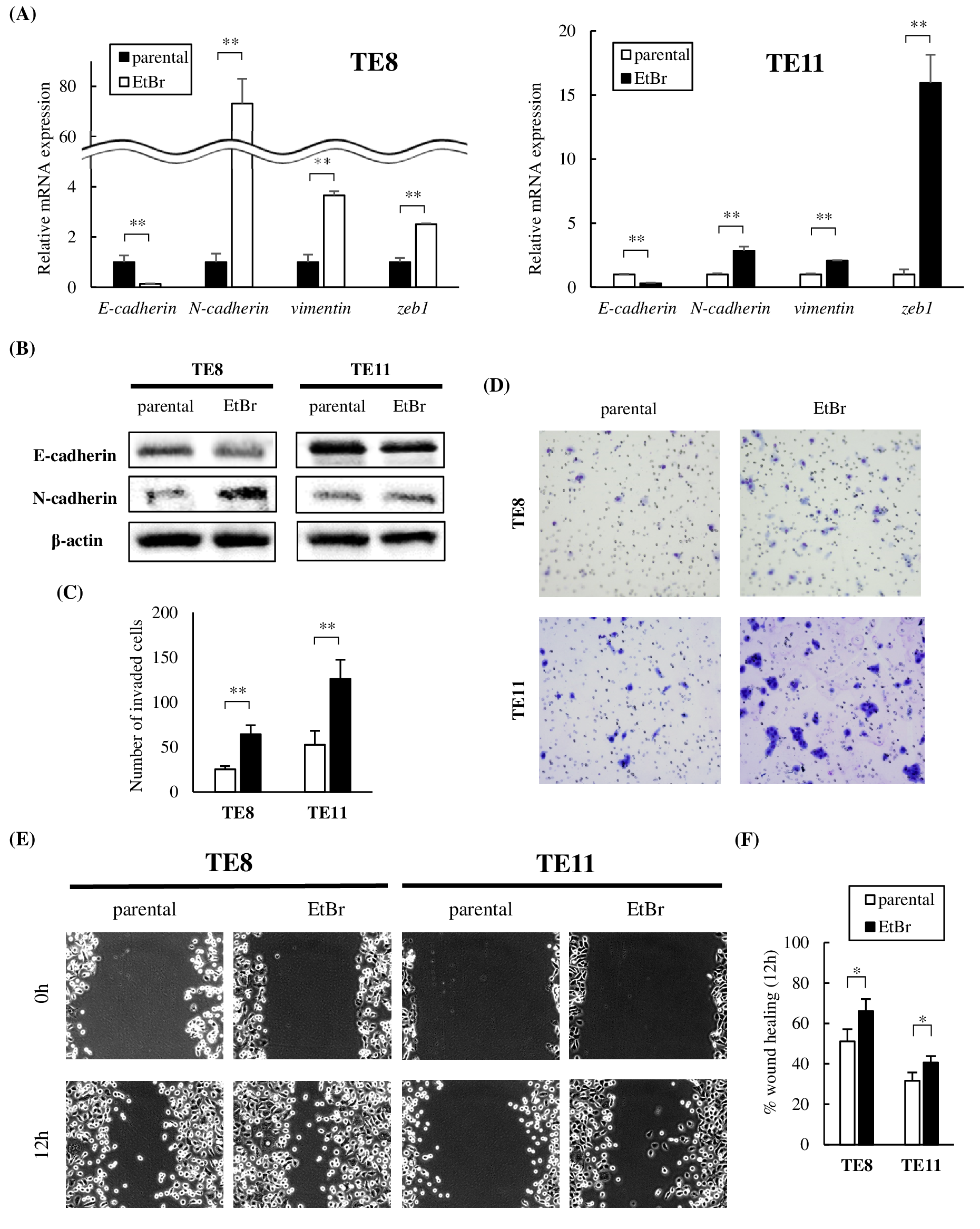

Supplement: S2 Fig — (A) The mRNA levels of four genes related to epithelial–mesenchymal transition were analyzed by qPCR. In both TE8 and TE11 cells, E-cadherin expression in mtDNA-depleted cells was significantly decreased, while N-cadherin, vimentin, and zeb-1 expression in mtDNA-depleted cells was significantly increased, compared with parental cells. (B) The protein levels of E-cadherin and N-cadherin were analyzed by immunoblotting. Compared with parental cells, in both TE8 and TE11 cells, E-cadherin protein level in mtDNA-depleted cells was decreased, while N-cadherin protein level in mtDNA-depleted cells was increased. (C, D) Both TE8 and TE11 mtDNA-depleted cells were significantly more invasive than parental cells (TE8: 64.3±10.0 vs 25.3±3.5; TE11: 126.0±21.4 vs 52.7±15.5, p<0.01). (E, F) The confluent monolayer of cells was scratched using a pipette tip, and the wounded area was measured at two time points (0 and 12 h). In both TE8 and TE11 cells, the wounded area was significantly decreased in mtDNA-depleted cells at 12 h, compared with parental cells (TE8: 66.0±6.0 vs 51.1±3.4%, p = 0.038; TE11: 40.6±3.2 vs 31.6±4.1%; p = 0.041). (TIF) [file pone.0193159.s002.tif]

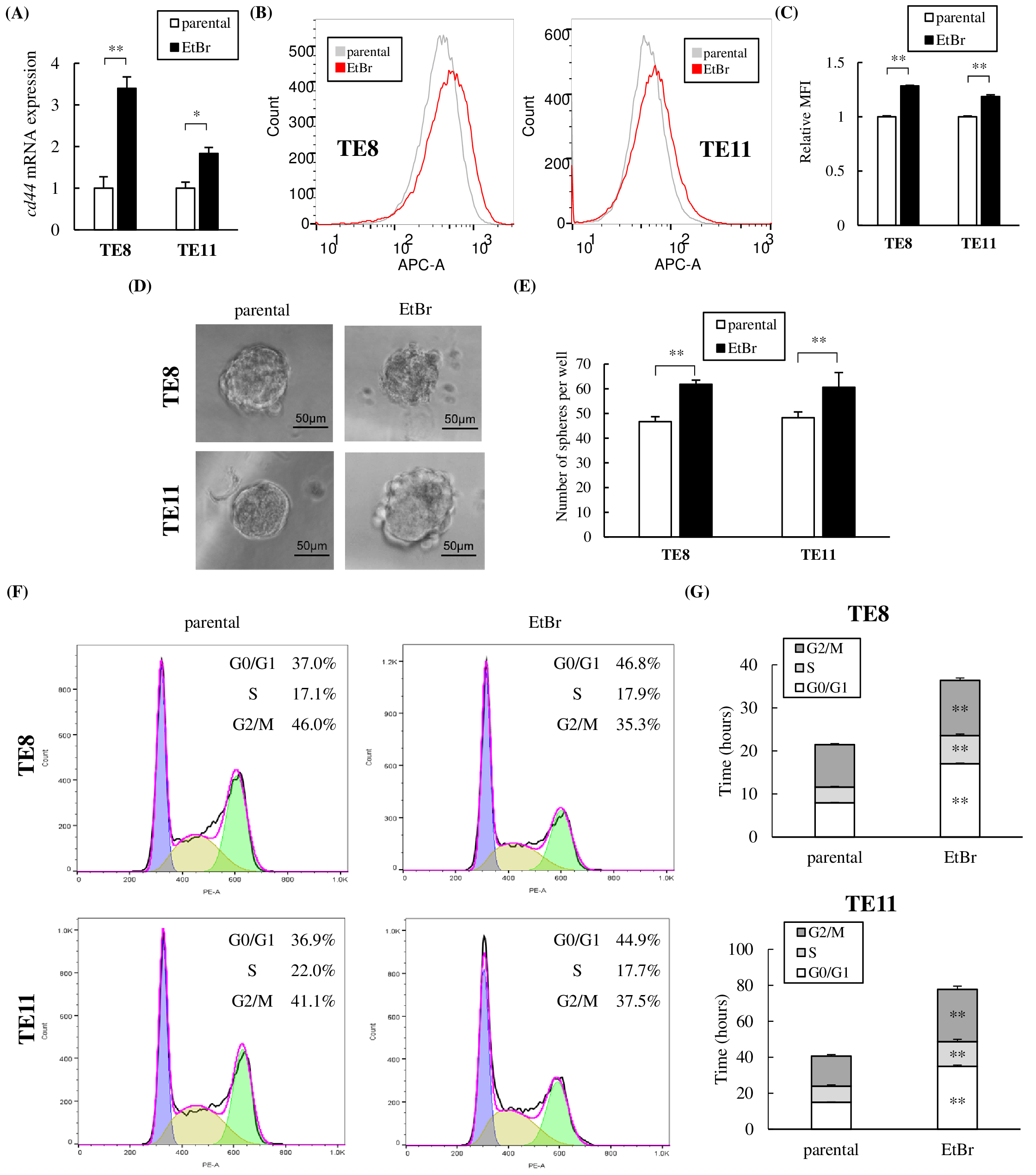

Supplement: S3 Fig — (A) In both TE8 and TE11 cells, cd44 expression of mtDNA-depleted cells was significantly increased compared with parental cells. (B, C) The protein expressions of CD44 were analyzed by flow cytometry using APC-CD44. MtDNA-depleted cells by EtBr treatment had higher protein expression of CD44 than parental cells. (D) Spheres formed by both TE8 and TE11 cells. (E) mtDNA-depleted cells formed significantly more spheres than parental cells (61.8±1.7 vs 46.7±2.0; TE11: 60.6±6.0 vs 48.3±2.3; p<0.01) (F, G) The duration in G0/G1 phase was significantly longer in mtDNA-depleted cells than in parental cells (TE8: 17.0±0.2 vs 7.9±0.1 h; TE11: 34.9±0.7 vs 15.0±0.2 h; p<0.01). (TIF) [file pone.0193159.s003.tif]
